# Impact of a training intervention on upper gastrointestinal endoscopy quality over time: Multicenter comparative cohort study

**DOI:** 10.1055/a-2526-0240

**Published:** 2025-03-14

**Authors:** Lieke Maria Koggel, Jole P.E. van Berlo, Fleur A. Indemans, Ruud W.M. Schrauwen, Marten A. Lantinga, Peter D. Siersema

**Affiliations:** 1 6034 Gastroenterology and Hepatology, Radboudumc, Nijmegen, Netherlands; 2 72489 Gastroenterology and Hepatology, Maasziekenhuis Pantein, Boxmeer, Netherlands; 3 97772 Gastroenterology and Hepatology, Bernhoven Hospital Location Uden, Uden, Netherlands; 4 Gastroenterology and Hepatology, Amsterdam Gastroenterolgy Endocrinology Metabolism, Amsterdam UMC, Amsterdam, Netherlands; 5 Gastroenterology and Hepatology, Erasmus MC University Medical Center, Rotterdam, Netherlands

**Keywords:** Endoscopy Upper GI Tract, Quality and logistical aspects, Training, Quality management, Performance and complications, Image and data processing, documentatiton

## Abstract

**Background and study aims:**

The European Society of Gastrointestinal Endoscopy (ESGE) and British Society of Gastroenterology (BSG) formulated performance measures to improve the detection rate for upper gastrointestinal (UGI) endoscopy. We aimed to assess adherence to and impact of training on adherence to performance measures for UGI endoscopy.

**Methods:**

In this multicenter, prospective, cohort study, endoscopists at three centers underwent 1-hour face-to-face training based on ESGE and BSG procedure performance measures (≥ 7-minute inspection time; photodocumentation of ≥ 10 anatomical landmarks + abnormalities; standardized terminology; biopsy protocols). A self-developed quality assessment score was used to assess diagnostic UGI endoscopies before (control group) and after (intervention group) training. The primary endpoint was improvement in overall quality score (percentage of the maximum score).

**Results:**

Of 1,733 consecutive UGI endoscopies, 570 were eligible for inclusion (mean patient age 60 years [standard deviation 15]; male 47%): 285 in the control group and 285 in the intervention group. Overall quality score increased from 60% before to 67% after the training intervention (difference 7%, 95% confidence interval [CI] 5–10, *P* < 0.001). Male patients (3.2%, 95% CI 0.7–5.7), alarming features (-3.1%, 95% CI -5.6 to -0.5), and endoscopist age (-0.4% increment per year, 95% CI -0.8 to -0.1) were associated with higher quality scores.

**Conclusions:**

Adherence to the ESGE and BSG procedure performance measures for UGI endoscopy persistently increased after a 1-hour face-to-face training intervention, suggesting that a simple training intervention tool can improve the quality of UGI endoscopy and potentially could prevent missed lesions.

## **Introduction**

Upper gastrointestinal (UGI) endoscopy is the gold standard for diagnosis and treatment of many UGI diseases. Nonetheless, the diagnostic yield of UGI endoscopy is limited because > 80% of UGI endoscopies do not change clinical management 1 . In contrast, up to 11% of UGI malignancies are missed during endoscopies performed up to 3 years before diagnosis, hampering early diagnosis, which is important to increase patient survival rates 2 .

The yield of UGI endoscopy is influenced by two elements. First, appropriate indications increase the likelihood of a clinically significant finding 3 . Second, quality of the endoscopy affects diagnostic performance. Due to lack of evidence about the association between quality indicators for UGI endoscopy and detection of pathology, there is no consensus on the optimal technique of examination to perform an UGI endoscopy. This contrasts with colonoscopy procedures for which international guidelines have been implemented regarding inspection time, photodocumentation, and biopsies, which in turn has improved (adenoma) detection rates of colonoscopies 4 5 6 .

Over the past years, different expert-reviewed performance measures for UGI endoscopy have been formulated by several societies, among them the European Society of Gastrointestinal Endoscopy (ESGE) and the British Society of Gastroenterology (BSG) 7 8 . Recommended indications can be divided into three categories: pre-procedure (e.g. fasting instructions), procedure (e.g. inspection time, photodocumentation, use of standardized terminology, and compliance with biopsy protocols) and post-procedure (e.g. Barrett registry). Although the scientific evidence is scarce, it is believed that implementation and repetitive training of these standards will lead to improvement in diagnostic performance of UGI endoscopy 7 .

Even though quality improvement is crucial to increase the yield of UGI endoscopy, studies evaluating the quality of UGI endoscopies are limited 9 10 11 . Adherence to performance measures for UGI endoscopy in daily clinical practice has only been assessed in a single-center setting in tertiary centers, although the majority of UGI endoscopies are performed in regional hospitals. Therefore, the aim of our study was to assess adherence to ESGE and BSG performance measures for UGI endoscopy in both an academic center as well as two regional hospitals, and to evaluate improvement after a training intervention.

## **Patients and methods**

### Study design and data collection

This multicenter, comparative cohort study was performed in three Dutch centers, including two regional hospitals (Bernhoven Hospital, Uden and Maashospital Pantein, Beugen) and one tertiary referral center (Radboud university medical center, Nijmegen). All endoscopists received a face-to-face interactive 1-hour training (between November 2021 and March 2022) on a selection of the ESGE and BSG procedural performance measures for UGI endoscopy. Afterward, the training material was made accessible to all endoscopists and summary posters were pinned up in every endoscopy suite ( **Appendix 1** ). Consecutive diagnostic UGI endoscopies performed by trained endoscopists in individual patients (e.g. no paired endoscopies) before (control group) and after (intervention group) the training intervention were retrospectively evaluated for adherence to performance measures by means of patient chart review, based on the endoscopy report and photodocumentation. Pre-procedure and post-procedure performance measures were excluded in this study because they are already structurally implemented in The Netherlands and not directly related to endoscopist performance. All centers used new-generation endoscopes and the same endoscopes were used during the course of this study. The academic center used RVC Clinical Assistant (Nexus Nederland) as the reporting system with standardized formats; the regional hospitals used free entry fields.

Records of UGI endoscopies, including pathology reports, patient characteristics, and clinical symptoms, were collected. Extracted data were manually entered into pre-defined digital case record forms using Castor Electronic Data Capture (Ciwit BV, Amsterdam, The Netherlands) 12 . Data entry was performed by two researchers (LK and JvB) who were trained in interpreting performance measures from patient charts. Both researchers registered data from UGI endoscopies at all participating centers for both measurement moments (before and after training intervention) to prevent administration bias. In case no consensus was reached about how to fill in an input field, the case was discussed with a third researcher, who is an experienced endoscopist (PS).

All diagnostic UGI endoscopies performed on adult patients (≥ 18 years old) were assessed for eligibility. We excluded: (1) endoscopies of patients who actively opted-out for chart review studies; (2) endoscopies of patients who received an UGI endoscopy in the last 36 months (ESGE only emphasizes complete performance of repeat endoscopy in case the prior endoscopy was performed > 36 months ago); (3) non-diagnostic endoscopies (e.g. for therapeutic or research indication); and (4) incomplete endoscopies (e.g. early termination due to patient intolerance or safety reasons, inability to visualize the whole UGI tract because of altered anatomy or stricture, inability to make a complete report due to technical reasons, or emergency setting). Formal medical ethical review was waived for this study (MREC Oost-Nederland, reference 2020–6693).

### Performance measures

**Fig. 1:**
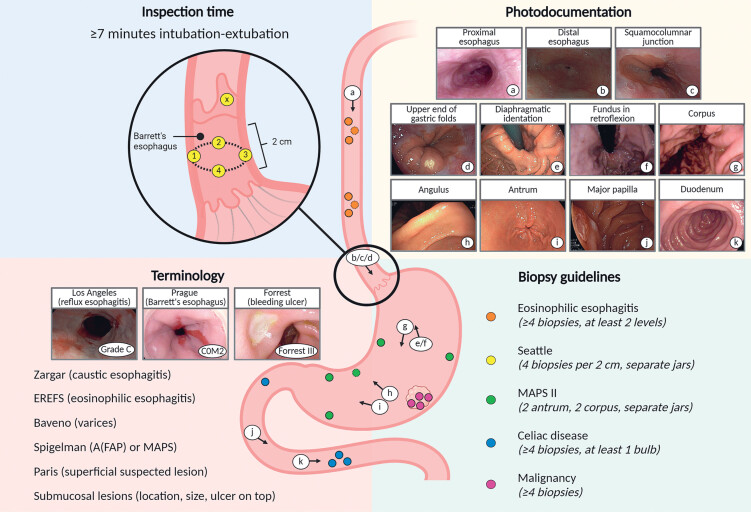
Performance measures for upper gastrointestinal endoscopy. Created in BioRender. Siersema, P. (2025) https://BioRender.com/y04k288

Training and assessment included procedural performance measures for UGI endoscopy based on a selection of ESGE and BSG performance measures, complemented by recommendations from local expert endoscopists ( Fig. 1 ) 7 8 . Performance measures comprised inspection time (≥ 7 minutes), photodocumentation (≥ 10 anatomical landmarks + all abnormalities), use of standardized terminology classifications (Los Angeles, Zargar, Prague, Forrest, Spigelman, Paris, Baveno, Endoscopic Reference Score [EREFS], and description of submucosal lesions) and compliance with biopsy protocols (Seattle, Management of Epithelial Precancerous Conditions and Lesions in the Stomach [MAPS] II, eosinophilic esophagitis, celiac disease, and suspected neoplasia). Inspection time was scored based on time between first and last photo due to missing intubation and extubation times as registered by endoscopy nursing staff in two of three centers. Photodocumentation of anatomical landmarks or abnormalities was also scored as positive in case of video documentation. All assessed UGI endoscopy performance measures and individual requirements for separate criteria can be found in **Appendix 1** .

### Quality score

The primary outcome was improvement in the total quality score, as measured by a new developed instrument ( **Appendix 2** ) because no quality score was yet available. This instrument has been developed, pilot-tested, and adapted through discussions among three expert endoscopists in the field of upper gastrointestinal endoscopy, all working at the academic center, on the results of a variety of endoscopy test cases until consensus about the total quality scores was reached. All participating centres agreed to the design and interpretation of the quality score.

For each item (inspection time, photodocumentation, standardized terminology, and biopsy protocols) a score of 0, 1, or 2 points could be achieved. For item inspection time, a score of 0 was assigned if inspection time was < 5 minutes, 1 if inspection time fell between 5 and 7 minutes, and 2 if the inspection time was ≥ 7 minutes. For photodocumentation, a score of 0 was given if there were less than six landmarks photographed or if photos of the abnormalities were not documented, 1 if six to nine landmarks and all abnormalities were photographed, and 2 if ≥ 10 landmarks and all abnormalities were photographed. Regarding standardized terminology, a score of 0 was given if no terminology classifications were used when applicable according to the guidelines, 1 if some but not all applicable terminology classifications were used, and 2 if all applicable terminology classifications were used correctly or if no terminology classification was used when not applicable. For biopsy guidelines, a score of 0 was assigned if no biopsies were taken when indicated or if biopsies were taken when not applicable, 1 if some but not all biopsies were taken as indicated, and 2 if all applicable biopsies were taken according to protocol or if no biopsies were taken when not indicated.

Quality score per UGI endoscopy was calculated as the total amount of achieved points/maximum to be obtained points x 100% and ranges between 0% and 100%. All UGI endoscopies could score the maximum number of points (8 points), also in case no pathology was found.

### Variables associated with higher quality scores

We determined variables associated with higher quality scores. Besides endoscopy characteristics, also the indication (because some indications predict a higher pathology rate) and setting in which the endoscopy was performed (due to for example time-pressure) could be associated with quality score outcome. Furthermore, patient characteristics could influence quality because the endoscopist might perform the endoscopy more thoroughly when the probability of certain pathology is higher (such as in case of a specific gender, age group, alarming features, positive family history, or risk factors such as obesity, alcohol use, and tobacco use). Therefore, variables included patient characteristics (gender, age, body mass index, alcohol use, smoking habits, American Society of Anesthesiologists [ASA] score, presence of alarming features, family history of UGI cancer, and performance of previous UGI endoscopy) and UGI endoscopy characteristics (indication, urgency, and day part of UGI endoscopy, sedation use, patient tolerance, and outcome) and endoscopist characteristics (gender, age, and experience defined as consultant vs. resident).

Outcome of UGI endoscopy was subdivided into no significant pathology, benign pathology, and (pre)malignant pathology (including *Helicobacter pylori* gastritis, gastric atrophy or intestinal metaplasia, Barrett’s esophagus [BE], duodenal dysplasia, and duodenal polyps/adenomas).

### Variables affecting improvement in quality scores

Variables included for analysis of their effect on improvement in quality scores after training were center, procedure characteristics (indication, urgency, sedation use, patient tolerance, and outcome) and endoscopist characteristics (person, gender, age, and experience defined as consultant vs. resident).

### Sample size

Sample size was calculated based on the primary outcome measure: improvement in total quality score. We used a database of 202 UGI endoscopies performed by 23 (trainee) endoscopists (mean of 14 UGI endoscopies per endoscopist) between October 2019 and February 2020 at the Radboud university medical center, Nijmegen, to estimate the mean quality score before intervention. Sample size was calculated using GPower 3.1.7 13 , assuming a mean quality score before intervention of 67% and a mean quality score after intervention of 72% (5% increase) with a standard deviation (SD) of 17%. The 1-β was set to 90% and the α to 5%, resulting in a total of 124 gastroscopies required. Because of the hierarchical structure of our study (UGI endoscopies nested within endoscopists), the sample size was multiplied by 4.6 to account for the estimated design effect based on the intracluster correlation coefficient of 0.15 and a mean of 25 UGI endoscopies per endoscopist. Based on this, 570 endoscopies were required, half of them before and half of them after the training intervention.

### Statistical analysis

Results were expressed as number and proportion or mean ± SD. Baseline characteristics were compared using Student’s *t* -test or chi-square test, as applicable. To measure the effect of the intervention on the mean quality score, a linear mixed model regression was used, because of the hierarchical structure of our data (UGI endoscopies nested within endoscopists). Interaction effects were determined to indicate the influence of a third variable on the difference in quality score between the before and after training group. To identify variables associated with adherence to the performance measures, a multivariable linear mixed model analysis was used. Variables with *P* < 0.2 in the univariable analysis were included in the multivariable analysis. A backwards model was used to stepwise eliminate the variables with the highest *P* value until all variables in the model had *P* < 0.05. Two-sided testing with *P* < 0.05 was considered significant. SPSS statistics version 27.0 (IBM Corp., Armonk, New York, United States) was used for all analysis.

## Results

**Fig. 2:**
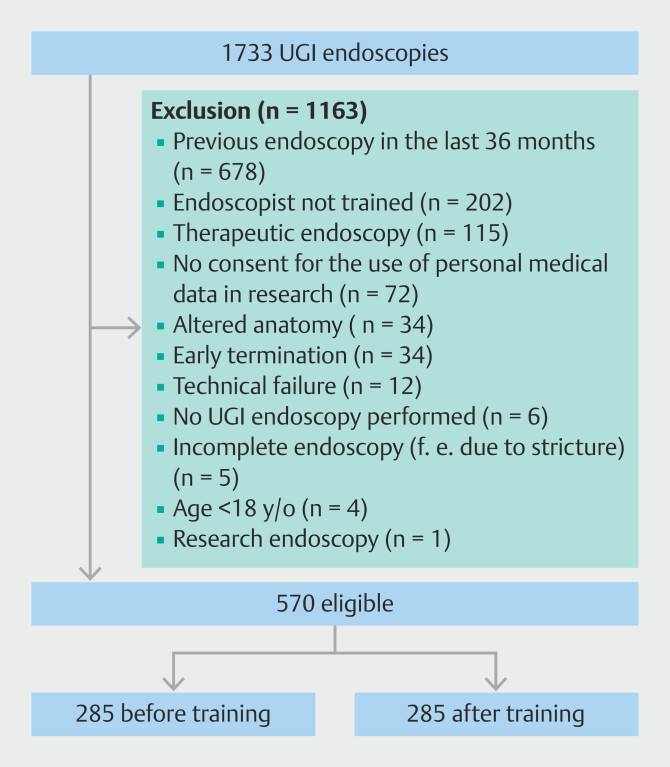
Endoscopy selection.

A total of 1,733 UGI endoscopies were evaluated of which 570 were included for analysis: 285 before training and 285 after training ( Fig. 2 ). UGI endoscopies were mainly excluded for the following reasons: previous UGI endoscopy in the last 36 months (n = 678), endoscopist not part of the training intervention (n = 202) or therapeutic interventions during endoscopy (n = 115). The 28 endoscopists performed a median of 19 UGI endoscopies per endoscopist (IQR 9 -24). Mean endoscopist age was 44 years (SD 9) with 57% being male. A majority of 82% was consultant vs. 18% resident. Data on UGI endoscopies were collected from 14 weeks (two centers) or 6 weeks (one center) before training to 6 weeks (one center) or 16 weeks (two centers) after training.

Mean patient age was 60 years (SD 15) with 47% being male ( Table 1 ). A total of 244 patients (43%) presented with alarming features (gastrointestinal bleeding, dysphagia, persistent vomiting, iron deficiency anemia, or unintended weight loss) and 49 (9%) had a family history of UGI malignancies. The main indications for UGI endoscopy were dyspepsia or reflux, anemia, and dysphagia in 200 (35%), 91 (16%), and 83 (15%) UGI endoscopies, respectively. Forty-five (8%) UGI endoscopies were performed in an urgent setting (< 24 hours). Almost all patients received a form of sedation (n = 478, 84%), mostly midazolam (n = 261, 46%) and topical pharyngeal anesthesia (n = 176, 31%) followed by procedural sedation and analgesia [PSA] or general anesthesia (n = 41,7%). No sedation was used in 55 patients (10%) and no data on sedation used were available for 37 patients (6%). Difference in baseline characteristics was only seen in the number of patients that underwent a previous (> 36 months) UGI endoscopy (n = 56 [20%] before training vs. n = 79 [28%] after training, *P* = 0.023). Benign or (pre)malignant pathology was found in 151 (27%) and 120 (21%) UGI endoscopies, respectively ( **Table 2, Appendix 2** ).

**Table:** **Table 1** Patient and procedure characteristics.

	All (n = 570)	Before training (n = 285)	After training (n = 285)	*P* value
**Patient characteristics**				
**Gender (male), n (%)**	266 (47)	137 (48)	129 (45)	0.502
**Age, mean (SD)**	60 (15)	60 (16)	60 (15)	0.792
BMI*, mean (SD)	26 (5)	26 (5)	26 (5)	0.322
Alcohol*, n (%)	243 (43)	120 (21)	123 (22)	0.894
Smoking*, n (%)	84 (15)	44 (15)	40 (14)	0.466
ASA*, n (%)				0.836
1–2	409 (72)	198 (70)	211 (87)	
3–4	68 (12)	32 (11)	36 (13)	
Family history of UGI malignancy*, n (%)	49 (9)	23 (8)	26 (9)	0.657
**Alarming features, n (%)**	244 (43)	126 (44)	118 (41)	0.498
**Previous (> 36 months) UGI endoscopy, n (%)**	135 (24)	56 (20)	79 (28)	0.023
Outcome previous UGI endoscopy*, n (%)				0.679
No significant pathology	56 (10)	21 (7)	35 (12)	
Benign pathology	33 (6)	15 (5)	18 (6)	
(Pre)malignant pathology	40 (7)	18 (6)	22 (8)	
**Procedure characteristics**				
Daypart of endoscopy*, n (%)				0.577
Morning	359 (63)	181 (64)	178 (63)	
Afternoon	210 (37)	103 (36)	107 (38)	
**Urgency of endoscopy, n (%)**				0.221
< 24 h	45 (8)	28 (10)	17 (6)	
24–72 h	21 (4)	11 (4)	10 (4)	
> 72 h	504 (88)	246 (86)	258 (91)	
Sedation*, n (%)				0.111
No sedation	55 (10)	34 (12)	21 (7)	
Topical pharyngeal anesthesia only	176 (31)	97 (34)	79 (28)	
Midazolam	261 (46)	122 (43)	139 (49)	
PSA or general anesthesia	41 (7)	19 (7)	22 (8)	
Patient tolerance*, n (%)				0.699
Good-fair	275 (48)	143 (50)	132 (46)	
Poor-very poor	45 (8)	22 (8)	23 (8)	
**Indication, n (%)**				0.314
Dyspepsia/reflux	200 (35)	101 (35)	99 (35)	
Anemia	91 (16)	45 (16)	46 (16)	
Dysphagia	83 (15)	42 (15)	41 (14)	
Hematemesis/melena	36 (6)	24 (8)	12 (4)	
Surveillance	33 (6)	15 (5)	18 (6)	
Abnormality on image	24 (4)	8 (3)	16 (6)	
Other	103 (18)	50 (18)	53 (19)	
**Outcome of endoscopy, n (%)**				0.686
No significant pathology	299 (53)	147 (52)	152 (53)	
Benign pathology	151 (27)	80 (28)	71 (25)	
(Pre)malignant pathology	120 (21)	58 (20)	62 (22)	
ASA; American Society of Anesthesiologists; BMI, body mass index; SD, standard deviation; UGI, upper gastrointestinal. *Missing: BMI n = 84, alcohol n = 105, smoking n = 84, ASA n = 93, family history of UGI malignancy n = 283, outcome previous UGI endoscopy n = 441, day part of endoscopy n = 1, sedation n = 37, patient tolerance n = 250.					

### Quality score

**Fig. 3:**
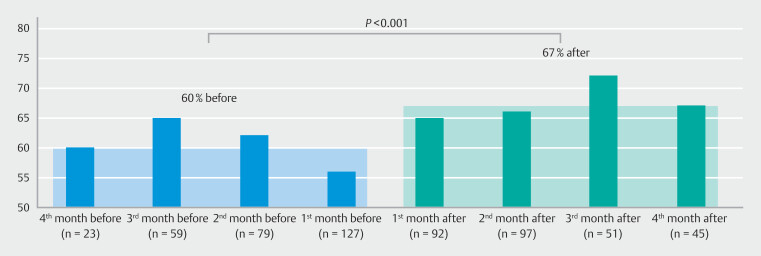
Mean quality score (percentage) before vs. after training.

Overall mean quality scores before vs. after training were 60% vs. 67% (difference 7%, 95% confidence interval [CI] 5–10, *P* < 0.001) ( Fig. 3 ). Improvement was seen in all centers with mean quality scores before vs. after training of 65% vs. 72% (difference 8%, 95% CI 3–12), 56% vs. 61% (difference 5%, 95% CI 1–9) and 59% vs. 68% (difference 9%, 95% CI 4–10), respectively, for centers one, two, and three. The majority of endoscopists showed improvement in quality score after training ( **Table 3, Appendix 2)** . No washout effect of the training intervention was seen over time during a follow-up up to 16 weeks ( Fig. 3 ).

**Fig. 4:**
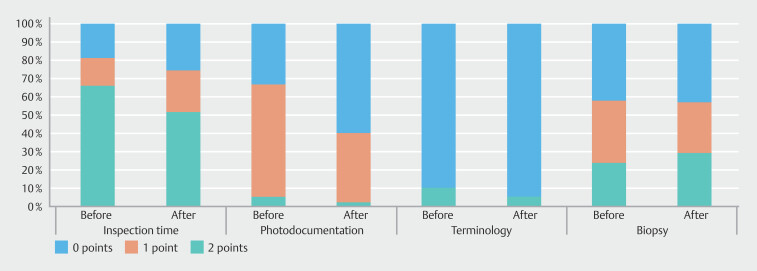
Quality score (number of points) per item before vs. after training Inspection time: 0 points (< 5 minutes), 1 point (5–7 minutes), 2 points (≥ 7 minutes). Photodocumentation: 0 points (< 6 landmarks* or no abnormalities), 1 point (6–9 landmarks* or no abnormalities), 2 points (≥ 10 landmarks* and all abnormalities). Terminology: 0 points (no use of standardized terminology ^†^ when applicable), 1 point (correct use of some but not all applicable standardized terminology**), 2 points (correct use of all applicable terminology** or no use when not applicable). Biopsy: 0 points (no biopsies taken according to protocol ^‡^ when indicated or taken when not indicated), 1 point (biopsies partly taken according to protocol ^‡^ ), 2 points (all biopsies taken according to protocol ^‡^ when indicated or not taken when not indicated). *Proximal esophagus, distal esophagus, squamocolumnar junction, upper end of the gastric folds, diaphragmatic indentation, retroflex fundus/cardia, corpus, angulus, antrum, duodenal bulb, distal duodenum, major papilla, all abnormalities. ^†^ Los Angeles, Zargar, Prague, Forrest, Spigelman, Paris, Baveno, EREFS classification and description of submucosal lesions (location, size, ulcer on top) ^‡^ Seattle, MAPS II, eosinophilic esophagitis, celiac disease, suspected neoplasia protocol

5 to 7, and < 5 minutes was seen in 19%, 15%, and 66% before training and 26%, 23%, and 52% after training, respectively ( Fig. 4 ).

Photodocumentation of (1) ≥ 10 anatomical landmarks and all abnormalities, (2) six to nine anatomical landmarks and all abnormalities or (3) fewer than six anatomical landmarks or no photodocumentation of abnormalities was performed in 33%, 61%, and 5% before training and 60%, 38%, and 2% after training, respectively. Anatomical landmarks that were most frequently not documented were the papilla (n = 476, 84%), angulus of the stomach (n = 193, 34%), proximal esophagus (n = 145, 25%), and corpus of the stomach (n = 100, 18%) ( Table 2 ).

**Table:** **Table 2** Quality score per item and related to the ESGE and BSG minimal target percentages.

Element	**Performance measures**	**ESGE minimum standard (%)**	**BSG minimum standard (%)**	** Overall (n = 570) **	**Control group (n = 285)**	**Intervention group (n = 285)**
**Inspection time**	**≥ 7 minutes intubation to extubation**	**≥ 90**	**n.a.**	**128/570 (23)**	**55/285 (19)**	**73/285 (26)**
**Photodocumentation**	**≥ 10 anatomical landmarks and all abnormalities**	**≥ 90**	**≥ 90** (any lesion)*	**266/570 (47)**	**95/285 (33)**	**171/285 (60)**
	Proximal esophagus	n.a.	n.a.	425/570 (75)	194/285 (68)	231/285 (81)
	Distal esophagus	n.a.	n.a.	549/570 (96)	271/285 (95)	278/285 (98)
	Z-line	n.a.	n.a.	547/570 (96)	270/285 (95)	277/285 (97)
	Upper gastric folds	n.a.	n.a.	543/570 (95)	268/285 (94)	275/285 (97)
	Diaphragm indentation	n.a.	n.a.	545/570 (96)	269/285 (94)	276/285 (97)
	Retroflex fundus	n.a.	n.a.	543/570 (95)	274/285 (96)	269/285 (94)
	Corpus of stomach	n.a.	n.a.	470/570 (83)	215/285 (75)	255/285 (90)
	Angulus of stomach	n.a.	n.a.	377/570 (66)	164/285 (58)	213/285 (75)
	Antrum of stomach	n.a.	n.a.	514/570 (90)	249/285 (87)	265/285 (93)
	Duodenum	n.a.	n.a.	555/570 (97)	274/285 (96)	281/285 (99)
	Papilla	n.a.	n.a.	94/570 (17)	32/285 (11)	62/285 (22)
	Abnormalities	n.a.	n.a.	564/570 (99)	281/285 (99)	283/285 (99)
**Standardized terminology**	** Correct use of all applicable terminology or no use when not applicable **	**n.a.**	**n.a.**	**528/570 (93)**	**257/285 (90)**	**272/285 (95)**
	Forrest classification	≥ 95	n.a.	8/16 (50)	3/8 (38)	5/8 (63)
	Prague classification	≥ 95	> 90	37/37 (100)	17/17 (100)	20/20 (100)
	Zargar classification	≥ 95	n.a.	0/0 (n.a.)	0/0 (n.a.)	0/0 (n.a.)
	Spigelman classification	≥ 95	n.a.	6/7 (86)	3/3 (100)	3/4 (75)
	Paris classification	≥ 95	> 90	0/13 (0)	0/7 (0)	0/6 (0)
	Baveno classification	≥ 95	n.a.	11/18 (61)	5/9 (56)	6/9 (67)
	Los Angeles classification	≥ 95	n.a.	76/85 (89)	31/39 (79)	45/46 (98)
	EREFS classification	n.a.	n.a.	1/1 (100)	0/0 (n.a.)	1/1 (100)
	Submucosal lesion description (location, size, ulcer on top)	n.a.	n.a.	2/10 (2)	0/6 (0)	2/4 (50)
**Biopsy protocols**	**All biopsies taken according to protocol when indicated or not taken when not indicated**	**n.a.**	**n.a.**	**240/570 (42)**	**119/285 (42)**	**121/285 (43)**
	Seattle protocol (4 per 2 cm, separate jars)	≥ 90	> 90	9/20 (45)	5/12 (42)	4/10 (40)
	Eosinophilic esophagitis (≥ 4, at least 2 levels) ^†^	n.a.	> 90	36/65 (55)	17/33 (52)	19/32 (59)
	MAPS II protocol (2 antrum, 2 corpus, separate jars) ^‡^	≥ 90	> 90	22/234 (9)	9/122 (7)	13/112 (12)
	Celiac disease (≥ 4, at least 1 bulb)	n.a.	> 90	81/204 (40)	39/98 (40)	42/106 (40)
	Suspected malignancy (≥ 4)	n.a.	> 90 (target biopsies)*	11/14 (79)	5/7 (71)	6/7 (86)
n.a., not applicable; values are n/N (%) unless otherwise defined. *Guideline advice (in parentheses) formulation differs from our scoring criteria. ^†^ BSG advises to take at least six biopsies from at least two different regions, we scored a minimum of four biopsies as mandatory; ^‡^ MAPS II guideline also advises taking biopsies according to protocol in case of a first endoscopy or in case of upper gastrointestinal symptoms, but that was not mandatory in our scoring. BSG, British Society of Gastroenterology; ESGE, European Society of Gastrointestinal Endoscopy; EREFS, Endoscopic Reference Score; MAPS, management of epithelial precancerous conditions and lesions in the stomach.						

Terminology classifications were correctly used, partly used, or not correctly used when applicable in 90%, 0%, and 10% before training and 95%, 0%, and 5% after training, respectively. Mostly correct used when applicable were the Prague classification in 37 of 37 UGI endoscopies (100%), the Los Angeles classification in 76 of 85 UGI endoscopies (89%), and the Spigelman classification in six of seven UGI endoscopies (86%). The lowest score was seen for the Paris classification with correct use when applicable in zero of 13 UGI endoscopies (0%), followed by correct description of submucosal lesions, the Forrest classification, and the Baveno classification in two of 10 (2%), eight of 16 (50%), and 11 of 18 (61%) UGI endoscopies, respectively ( **Table 4, Appendix 2** ).

Biopsies were correctly taken according to protocol or not taken when not indicated, partly taken, or not taken when indicated in 42%, 34%, and 24% before training and 43%, 28%, and 29% after training, respectively. Biopsies were correctly taken according to protocol when indicated for suspected malignancy in 11 of 14 UGI endoscopies (79%), eosinophilic esophagitis in 36 of 65 UGI endoscopies (55%), Seattle protocol in nine of 20 UGI endoscopies (45%), celiac disease in 81 of 204 UGI endoscopies (40%), and MAPS II protocol in 22 of 234 UGI endoscopies (9%). Some biopsies were taken but not completely according to protocol when indicated in 161 of 234 UGI endoscopies (69%) for the MAPS II protocol, 44 of 204 (22%) for celiac disease, and 10 of 65 (15%) for eosinophilic esophagitis ( **Table 5, Appendix 2** ).

### Sensitivity analysis inspection time

In one center (n = 190), not only the time of the first and last photo was documented, but also the exact time of intubation and extubation was scored by endoscopy nurses. When comparing these, mean inspection time based on photodocumentation was 2 minutes and 18 seconds less than the mean inspection time based on intubation-extubation (5:01 vs. 7:19, respectively). As a result of using inspection time based on photodocumentation instead of intubation and extubation, the number of points achieved for the item inspection time decreased from 1.19 (SD 0.94) to 0.68 (SD 0.83) and mean total quality score within this center decreased from 70 (SD 18) to 64 (SD 19). Mean quality score before training vs. after training were 66% and 77%, respectively (difference 9%, 95% CI 5–14, *P* < 0.001).

### Variables associated with higher quality scores

Variables with ≥ 10% missing (body mass index, alcohol use, smoking, ASA score, family history of UGI malignancies and patient tolerance) or not fully registered data (prior UGI endoscopy, outcome of prior UGI endoscopy) were excluded.

Variables associated with higher quality scores were male gender of the patient (3.2%, 95% CI 0.7–5.7), alarming features (-3.1%, 95% CI -5.6 to -0.5), and age of endoscopist (-0.4% increment per year, 95% CI -0.8 to -0.1) ( **Table 6, Appendix 2** ).

### Variables affecting improvement in quality scores

No significant effect of the variables on improvement in quality score after training was seen ( **Table 3, Appendix 2** ).

## Discussion

This comparative cohort study showed suboptimal adherence to the procedural ESGE and BSG performance measures for UGI endoscopy with an overall quality score of 60% which improved statistically significant to 67% after a 1-hour training intervention. The effect persisted up to at least 4 months follow-up. Characteristics of individual endoscopists did not predict improvement in quality scores. Higher overall quality scores for UGI endoscopy were attributed to patient/setting factors such as male patients, absence of alarming features, and younger age of the endoscopist.

Previous studies that analyzed the effect of UGI training interventions similarly reported improvement in adherence to quality indicators 9 10 11 . When comparing our intervention with prior studies, a key differentiator is our use of the ESGE and BSG performance measures, which had not yet been published at the start of two prior studies 9 10 . Also, we incorporated the findings of a pilot study conducted in the same population into the training as generalized feedback on the current performance status, which only one of the prior studies had implemented 10 .

Although slightly different criteria for quality scoring were used in the prior studies, some comparisons can be made. A higher rate of adequate inspection time (≥ 7 minutes) was previously reported (80% 11 in a prior study vs. 19% in our study before and 84% 11 vs. 26% after intervention). This could potentially be explained by the fact that we used photodocumentation times as a proxy for absence of actual intubation-extubation times. In contrast, our study showed higher complete photodocumentation rates (33% in our study vs. < 1% in prior studies 10 11 before and 60% vs. 3% to 76% 10 11 after intervention). As for use of terminology classifications, comparable high rates of correct use were reported (around 50%-88% 10 11 in prior studies vs. 90% in our study before and around 80% to88% 10 11 vs. 95% after intervention), suggesting an overall sufficient implementation of individual classification systems. Adherence to biopsy protocols was comparable (33% 10 in a prior study vs. 42% in our study before and 33% 10 vs. 43% after intervention) but leaves room for improvement. Although scoring details for use of different jars per region of MAPS guideline biopsies was not always clear, lower compliance with the MAPS guideline in our study (9% vs. 52%-91% 11 ) may also be explained by low prevalence of gastric carcinoma in The Netherlands which results in local endoscopists questioning the necessity for MAPS biopsies 14 . Furthermore, moderate adherence to the eosinophilic esophagitis biopsy protocol was comparable to prior studies (55% vs. 56%-76% 11 ) whereas celiac disease biopsies were performed less accurately in our study (50% vs. 89–98% 11 ). Potentially, the lower likelihood of celiac disease and eosinophilic esophagitis in elderly patients presenting with anemia or dysphagia may cause endoscopists to not perform biopsies according to protocol because they mainly focus on ruling out malignancies instead. Moreover, biopsy scores may have been underestimated because prior exclusion of celiac disease was not taken into account, which could justify not taking biopsies.

The association between higher quality scores and younger endoscopists seems in line with a prior study in which residents scored higher than specialists on, among others, lesion description (93% vs. 85%, respectively) 10 . Although the same learning potential was seen regardless of endoscopist age, overall higher scores by younger endoscopist may reflect increasing emphasis on quality of care within the training program. Moreover, more experienced endoscopists may diverge from protocols based on thoughtful consideration of potential benefits and disadvantages. With an increasing focus on value-based care, it is questionable whether overruling protocols results in an enhanced proposition or reduces quality of care. However, care must be taken to avoid rigidity in routine practice and lack of awareness about the most recent guidelines. An association with higher-quality endoscopy was also found in male patients. Although no specific data on the effect of patient gender in relation to quality performance of endoscopies are known, gender bias in favor of male patients has been reported as a general concern throughout healthcare 15 . Furthermore, lower quality scores were observed in patients presenting with alarming features, which could be explained by time-sensitive emergency settings. Yet the alarming features could contribute to a hyperfocus on outcomes that are associated with these symptoms, thereby hindering performance of a thorough diagnostic endoscopy 16 . In case of pathologic findings during endoscopy, more domains of the quality score (terminology, biopsy protocols) need to be met, potentially facilitating lower scores due a higher risk of score deduction, for example, in the case of incomplete biopsies. Because it is known that more pathology is found in patients with alarming features, this could influence the results.

Whether pathology detection actually improves when the (mainly expert-based) performance measures are met could not be assessed in our study due to a relatively small number of inclusions. The only prior study that assessed ESGE and BSG performance measures for UGI endoscopy in a tertiary hospital showed an increased diagnostic yield for *H. pylori* and BE detection, suggesting that a training intervention could improve detection of premalignant pathology 11 . For the terminology classifications and biopsy protocols, weak to moderate evidence is available that substantiates its added value 8 17 . Evidence for the best cut-off value regarding inspection time and direct benefits of photodocumentation is limited for UGI endoscopy, resulting in uncertainty regarding the importance of full compliance. Only two Asian studies have been performed focusing on overall inspection time of UGI endoscopy with cut-off times of 5 and 7 minutes 18 19 . This contrasts with colonoscopies, for which multiple studies led to a recommendation for a withdrawal time of 6 to 10 minutes to improve (adenoma) detection rate 4 . Extended photodocumentation has shown to improve early lesion detection in studies performed only in countries with a high incidence of gastric cancer 20 . Although evidence for photodocumentation in countries with low prevalence of gastric carcinomas is lacking, it is believed that systematic documentation improves mucosal inspection because it encourages mucosal cleaning, offers the opportunity to examine still images, and stimulates complete examination.

A strength of this study is implementation of a training intervention in a real-world setting reflecting clinical practice. Moreover, data were collected from multiple centers including non-university hospitals, which improves generalizability of the results, recognized guidelines were used to score quality of UGI endoscopy, and the sample size was calculated based on a pre-conducted pilot study. A notable limitation is utilization of a score instrument that has not been formally validated. However, because no validated score instrument is available as of now, we carefully drafted and thoroughly tested this new instrument to ensure the best possible assessment and made it as feasible and generalizable as possible to facilitate standardized monitoring. Second, the researchers who filled in the data and the endoscopists were not blinded. Despite the possibility that the latter could have introduced performance bias, periodic audits are known to enhance performance and, therefore, are desirable when implementing training interventions 21 . Third, retrospective assessment of individual scoring items may have led to measurement bias such as underestimation of inspection time because we had to use photodocumentation times as the best available proxy. However, sensitivity analyses were performed, and this underestimation only affected the overall quality score and not improvement after training intervention because both before and after measurements were affected by this limitation. Because endoscopists were aware of quality assessment of their endoscopies after the training intervention, which was not the case for endoscopies before the training session, the Hawthorne effect could play a role in improvement in quality scores after training and could have resulted in overestimation of improvement in the quality score. Also, a large number of endoscopies were excluded. Although this could have led to selection bias, this risk is thought to be minimal. A substantial number of endoscopies were excluded because of a prior endoscopy < 36 months. Most of these endoscopies were performed in the academic center which was also a referral center for BE. Therefore, most patients who underwent UGI endoscopy in this center were referred based on findings from a prior UGI endoscopy in a non-academic center and had to be excluded because complete inspection (and therefore eligibility for use of the quality score) was not deemed necessary. Moreover, some of the endoscopists performed only a limited number of endoscopies. To minimalize risk of bias, we took this into account in the sample size and statistical analysis by correcting for endoscopies nested within endoscopists. Last, the relatively short follow-up limited reliable assessment of a washout effect of the training intervention, although at least 4 months were evaluated.

We would advocate regulated annual monitoring of the quality of UGI endoscopy using a quality score including personalized feedback as has already been shown effective in colonoscopy 21 supplemented by structural training, made available to all endoscopists. It should be discussed whether a standardized training intervention should be mandatory for starting endoscopists and endoscopists with scores below a predefined threshold and repeated periodically until a sufficient score is achieved. In general, our data suggest that special attention should be paid to older endoscopists (> 50 years old) and endoscopies in female patients and patients with alarming features. Before implementation of the quality score, it needs to be validated. Furthermore, use of lesion detection systems and automation of processes is encouraged to optimize performances and facilitate quality monitoring.

## Conclusions

In conclusion, improvements need to be made in performance of UGI endoscopy in current clinical practice so that each endoscopy will be performed according to a high-quality standard regardless of indication or circumstances. A 1-hour training intervention seems to be an effective tool to increase adherence to UGI endoscopy performance measures, thereby potentially increasing the pathology detection rate.

## Supplementary Material

Supplementary Material Zusatzmaterial

Supplementary Material Zusatzmaterial
