# Supplementary material for: Impact of a training intervention on upper gastrointestinal endoscopy quality over time: Multicenter comparative cohort study
Source: Endosc Int Open. 2025 Mar 14;13:a25260240. doi: 10.1055/a-2526-0240 (PMC11922177; doi:10.1055/a-2526-0240)
Supplement: Supplementary file 1 — Supplementary Material [file 10-1055-a-2526-0240_25294060.pdf]

IMPlementation of quality standaRds for uppEr gaStrointestinal endoScopy

IMPRESS study - training

Radboudumc

Inspection time – Photodocumentation – Terminology – Biopsy

Inspection time

Radboudumc

Inspection time – Photodocumentation – Terminology – Biopsy

Inspection time - background

Teh et al[1]: Prospective, ±800 gastroscopies, 2010
≥7 min high-risk laesies OR 2.50, 95% CI 1.52-4.12
≥7 min neoplasia OR 3.42, 95% CI 1.25-10.38

Kawamura et al[2]: Retrospective, ±16.000 gastroscopies, 2010-2015
5-7 min neoplasia OR 1.90, 95% CI 1.06-3.40
≥7 min neoplasia OR 1.89, 95% CI 0.98-3.64

1. Teh JL, Tan JR, Lau LJF, et al. Longer examination time improves detection of gastric cancer during diagnostic upper gastrointestinal endoscopy. *Clin Gastroenterol Hepatol* 2015; 13: 480–487.e2.
2. Kawamura T. et al. Examination time as a quality indicator of screening upper gastrointestinal endoscopy for asymptomatic examinees. *Dig endosc.* 2017 Jul;29(5):569-575.

Radboudumc

Inspection time – Photodocumentation – Terminology – Biopsy

Inspection time - recommendation

Guideline	Advice
ESGE	≥ 7 minutes
BSG-AUGIS	Should be recorded for surveillance procedures
ASG/ASGE	-

Recommendation: ≥ 7 minutes (intubation-extubation)

Radboudumc

Inspection time – Pilot results

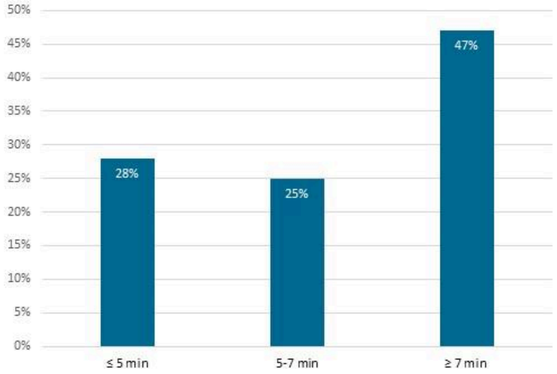

Radboudumc

Photodocumentation

Radboudumc

Inspection time – **Photodocumentation** – Terminology – Biopsy

Photodocumentation - Background

Indirect quality indicator for careful inspection of the digestive lumen

Systemic alphanumeric coded endoscopy (SACE)[3]
→ high frequency of gastric neoplasia (2.8% overall)

Ampulla photodocumentation[4]
→ higher neoplasia detection rate ($R = 0.57$, $p = 0.03$ / OR 1.33, 95%CI 1.03 - 1.70)

3. Machaca Quea NR, Emura F, Barreda Bolaños F, Salvador Arias Y, Arévalo Suárez FA, Piscoya Rivera A. Effectiveness of systematic alphanumeric coded endoscopy for diagnosis of gastric intraepithelial neoplasia in a low socioeconomic population. Endosc Int Open. 2016 Oct;4(10):E1083-E1089. doi: 10.1055/s-0042-115408. PMID: 27747283; PMCID: PMC5063642.
4. Park JM, Lim CH, Cho YK, et al. The effect of photo documentation of the ampulla on neoplasm detection rate during esophagogastroduodenoscopy. *Endoscopy* 2019; 51: 115–124.

Radboudumc

Inspection time – **Photodocumentation** – Terminology – Biopsy

Photodocumentation - Recommendation

Guideline	Advice		
ESGE	At least one representative photo of ≥ 10 of the following landmarks + all abnormalities		
	Proximal esophagus<br>Distal esophagus<br>Squamocolumnar junction<br>Upper end of the gastric folds<br>Diaphragmatic identation	Fundus in retroflexion<br>Corpus<br>Angulus<br>Antrum	Major papilla<br>Duodenum
BSG-AUGIS	Relevant anatomical landmarks + any detected lesions		
ASGE	Abnormalities		

Recommendation: at least 10 landmarks according to ESGE + all abnormalities

Radboudumc

Inspection time – **Photodocumentation** – Terminology – Biopsy

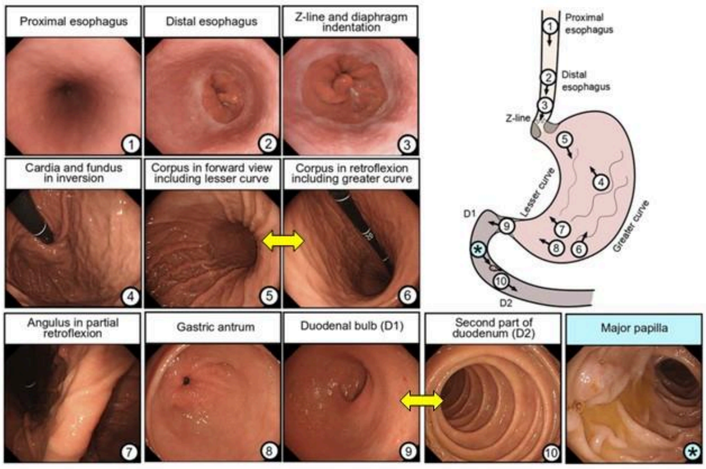

5. Januszewicz, W., & Kaminski, M. (2020). Quality indicators in diagnostic upper gastrointestinal endoscopy. *Therapeutic Advances in Gastroenterology*, *13*, 175628482091669

Radboudumc

Inspection time – **Photodocumentation** – Terminology – Biopsy

Photodocumentation – Pilot results

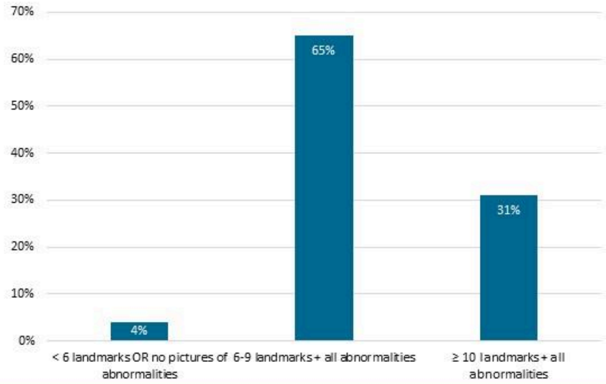

Radboudumc

Thieme

Inspection time – Photodocumentation – **Terminology** – Biopsy

Terminology

Radboudumc

Inspection time – Photodocumentation – **Terminology** – Biopsy

Terminology – Forrest

What? Forrest classification

When? Bleeding ulcer (currently or recently, gastric/duodenal)

Why? Predictor of rebleed

Classification		Rebleed risk
Ia	Arterial bleeding	90-100%
Ib	Oozing	80-85%
IIa	Visible vessel	40-50%
IIb	Adherent clot	20-30%
IIc	Flat pigmentation	5%
III	Clean base	≤ 3%

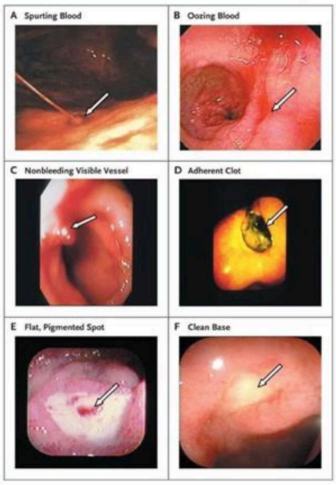

Radboudumc

Inspectieijd – Photodocumentation – **Terminology** – Biopsy

Terminology – Prague

What? Prague classification
When? Barret's esophagus (C≥1 or M≥1)
Why? Related to neoplasia rate

C	The distance from the top of the gastric folds to the most proximal extent of the circumferential involvement of the BE
M	The distance from the top of the gastric folds to the most proximal extent of BE

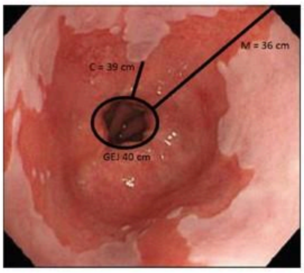

Radboudumc

Inspection time – Photodocumentation – **Terminology** – Biopsy

Terminology – Zargar

What? Zargar
When? Caustic esophagitis
Why? Predictor of morbidity

Grade 0	Normal mucosa
Grade I	Edema and erythema
Grade IIA	Hemorrhage, erosions, blisters, superficial ulcers
Grade IIB	Circumferential lesions
Grade IIIA	Focal deep gray or brownish-black ulcers
Grade IIIB	Extensive deep gray or brownish-black ulcers
Grade IV	Perforation

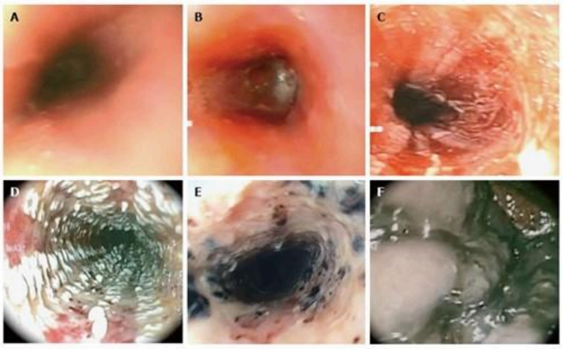

Radboudumc

Inspection time – Photodocumentation – **Terminology** – Biopsy

Terminology – Spigelman

What? Spigelman classification
When? Duodenal adenomas in patients with known (A)FAP or MAPS
Why? Predictor of malignancy rate → surveillance interval

**Criteria**	**Points**		
	**1**	**2**	**3**
**Polyp number**	1-4	5-20	>20
**Polyp size (mm)**	1-4	5-10	>10
**Histology**	Tubular	Tubulovillous	Villous
**Dysplasia**	Mild	Moderate	Severe

**Stage**	**Score**	**Surveillance interval**
Stage 0	0 points	5 years
Stage I	1-4 points	5 years
Stage II	5-6 points	3 years
Stage III	7-8 points	1 year
Stage IV	9-12 points	6 months

Radboudumc

Inspection time – Photodocumentation – **Terminology** – Biopsy

Terminology – Paris

What? Paris classification
When? Superficial suspected (pre)malignant lesions. Excl: fundic gland.
Why? Assessment of endoscopic resectability

Grade Ip	Predunculated
Grade Ips	Subpedunculated
Grade Is	Sessile
Grade IIa	Flat elevation of mucosa
Grade IIa+c	Flat elevation with central depression
Grade IIb	Flat mucosal change
Grade IIc	Mucosal depression
Grade IIc+IIa	Mucosal depression with raised edge
Grade III	Excavated
Grade IIc+III	Mucosal depression with raised edge and excavation

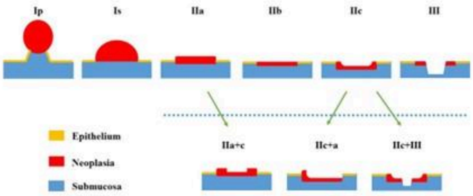

Radboudumc

Terminology – Baveno

What? Baveno
When? Varices
Why? Predictor of (re)bleed

Esophageal varices	Small (<5mm) or medium/large (≥5mm)<br>Red signs (red wale, cherry red spot, white nipple)
Gastric varices	Sarin classification
Portal gastropathy	Yes/no

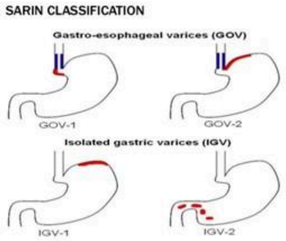

Sarin classification	
Gastro-oesophageal varices type 1 (GOV-1)	Continuation of oesophageal varices into the lesser curvature
Gastro-oesophageal varices type 2 (GOV-2)	Oesophageal and fundal varices are present in continuity with the greater curvature
Isolated gastric varices type 1 (IGV-1)	Fundal varices are present in the cardia in the absence of oesophageal varices
Isolated gastric varices type 2 (IGV-2)	Fundal varices present in the stomach outside of cardio-fundal region or first part of duodenum (IGV2)

Radboudumc

Terminology – Los Angeles

What? Los Angeles
When? Erosive esophagitis
Why? Related to severity of acid exposure

Grade A	One or more mucosal break < 5 mm that does not extend between the tops of two mucosal folds
Grade B	One or more mucosal break ≥ 5 mm that does not extend between tops of two mucosal folds
Grade C	One or more mucosal break that is continuous between the tops of two or more mucosal folds but that involves <75% of the circumference
Grade D	One or more mucosal break that involves ≥75% of the esophageal circumference

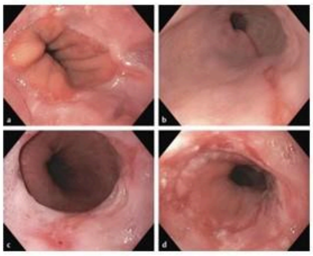

Radboudumc

Thieme

Inspection time – Photodocumentation – **Terminology** – Biopsy

Terminology – EREFS

What? EREFS
When? Eosinophilic esophagitis
Why? Predictor of severity

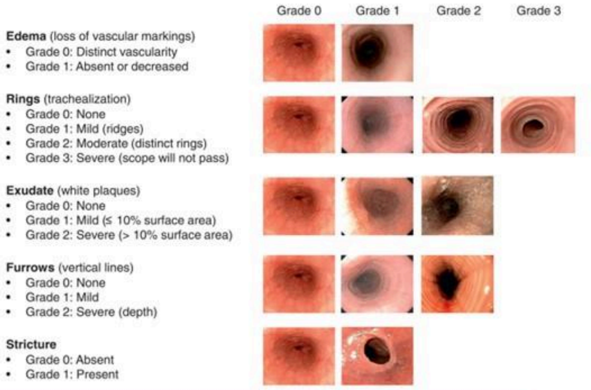

Radboudumc

Inspection time – Photodocumentation – **Terminology** – Biopsy

Terminology – Submucosal lesion

What? Location, size and ulcer on top *(pillow sign, delle, aspect of mucosa)*
When? Submucosal lesions
Why? Complete reporting for possible follow-up

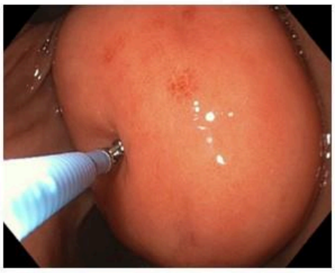

Radboudumc

Inspection time – Photodocumentation – **Terminology** – Biopsy

Terminology – pilot results

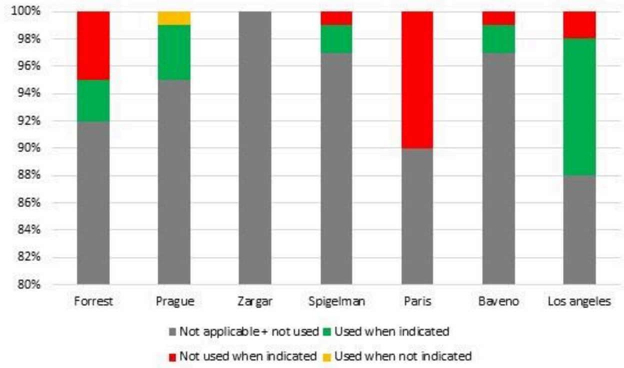

Radboudumc

Inspection time – Photodocumentation – Terminology – **Biopsy**

Biopsy

Radboudumc

Biopsy - Seattle

What? 4-quadrant biopsy every 2 cm, separate jar per level
Suspected lesion: targeted biopsies in separate jar

When? Barrett's esophagus (C≥1)

Why? Low-grade dysplasia:
- 18.9% Seattle vs.
- 1.6% nonsystematic biopsy sampling (P<0.001)

High-grade dysplasia:
- 2.8% Seattle vs.
- 0% nonsystematic biopsy sampling (P=0.03)[6]

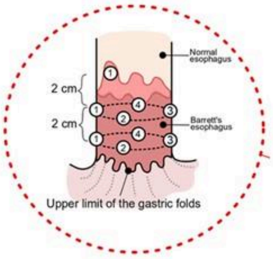

6. Abela, J., Going, J., Mackenzie, J. et al. Systematic Four Quadrant Biopsy Detects Barrett's Dysplasia In More Patients Than Nonsystematic Biopsy. The American Journal Of Gastroenterology, (2008) 103(4), 850 855

Radboudumc

Inspection time – Photodocumentation – Terminology – **Biopsy**

Biopsy – Eosinophilic esophagitis

What? ≥ 4 biopsies*, 2 different levels

When? Dysphagia or food occlusion without alternative cause

Why? 1 biopsy sensitivity 5%
> 5 biopsies sensitivity 100%[7]

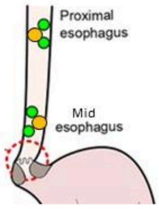

7. N. Gonsalves, M. Policarpio Nicolas, Q. Zhang, et al. Histopathologic variability and endoscopic correlates in adults with eosinophilic esophagitis. Gastrointest Endosc, 64 (2006), pp. 313 319

*Guideline deviation: BSG advises to take at least six biopsies from at least two different regions, we scored a minimum of four biopsies as mandatory

Radboudumc

Inspection time – Photodocumentation – Terminology – **Biopsy**

Biopsy – MAPS II

What? 2 biopsies antrum + 2 biopsies corpus (both greater + lesser curvature), separate jars

When?* Endoscopic features of gastric atrophy
Endoscopic features or intestinal metaplasia
Screening for *H. pylori*
Screening intestinal metaplasia

Why? Predictor of malignancy rate

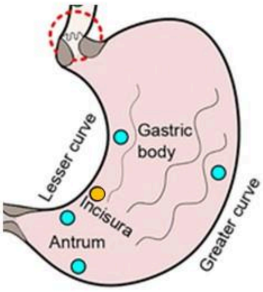

Exclusion incisura biopsy: 30-35% high-risk→ low-risk[8]

8. Isajevs S, Liepniece-Karele I, Janciauskas D et al. The effect of incisura angularis biopsy sampling on the assessment of gastritis stage. Eur J Gastroenterol Hepatol 2014; 26: 510 – 513

*Guideline deviation: MAPS II guideline also advises to take biopsies according to protocol in case of a first endoscopy or in case of upper gastrointestinal symptoms, this was not mandatory in our scoring

Radboudumc

Inspection time – Photodocumentation – Terminology – **Biopsy**

Biopsy – Celiac disease

What? ≥ 4 biopsies, at least 1 from bulb*

When? Suspected celiac disease (anemia, malabsorption, diarrhea, unintented weight loss, postprandial pain, meteorism, suggestive blood test)

Why? ≥4 biopsies: OR 3.7 (95% CI 2.9-4.7) Marsh III[9]
9-14% of CD villous atrophy in bulb alone[10]

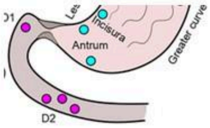

9. Lebwohl, B., Kapel, R, Neugut, A. et al. Adherence to biopsy guidelines increases celiac disease diagnosis. Gastrointestinal Endoscopy (2011), 74(1), 103-109.
10. Evans, K., Aziz, I., Cross, S. et al.. A Prospective Study of Duodenal Bulb Biopsy in Newly Diagnosed and Established Adult Celiac Disease. American Journal Of Gastroenterology (2011), *106*(10), 1837-1742

*Guideline deviation: if region of biopsies was unknown but the minimum of four biopsies were taken, it was scored as according to protocol

Radboudumc

Inspection time – Photodocumentation – Terminology – **Biopsy**

Biopsy - Malignancy

When? Suspected neoplasia

What? ≥ 4 biopsies*

Why?

	EsophagusCa[11, 12]	GastricCa[12]
1 biopsy	93%	
2 biopsies	95.8%	
4 biopsies	97.9%	95%
6 biopsies	100%	
7 biopsies		>98%

11. Lal N, Bhasin DK, Malik AK, *et al.* Optimal number of biopsy specimens in the diagnosis of carcinoma of the oesophagus. *Gut* 1992;33:724–6.
12. Graham DY, Schwartz JT, Cain GD, *et al.* Prospective evaluation of biopsy number in the diagnosis of esophageal and gastric carcinoma. *Gastroenterology* 1982;82:228–31.

*Guideline deviation: BSG advises to take at least six biopsies, we scored a minimum of four biopsies as mandatory

Radboudumc

Inspection time – Photodocumentation – Terminology – **Biopsy**

Biopsy – Pilot results

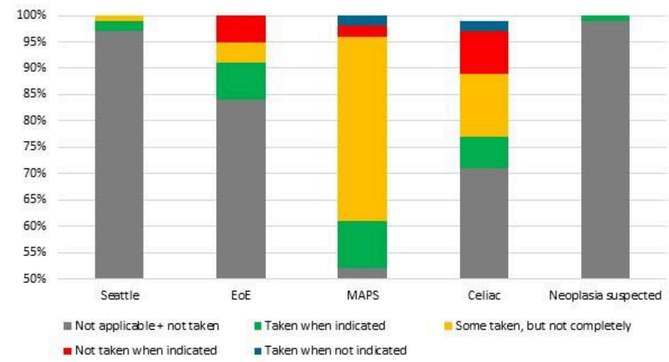

Radboudumc

Thieme

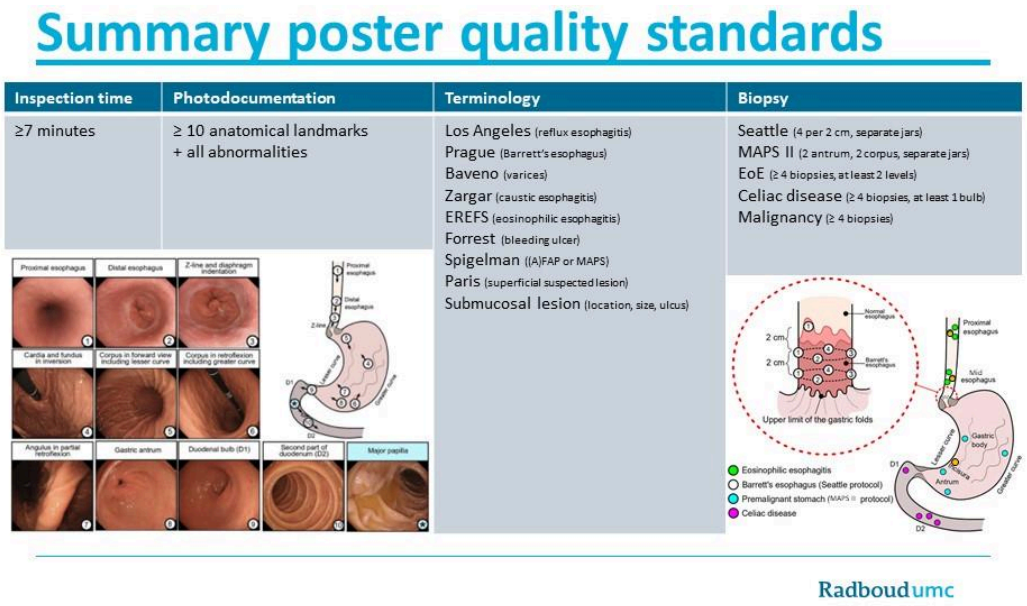
Summary poster quality standards
Inspection time
Photodocumentation
Terminology
Biopsy
≥7 minutes
≥ 10 anatomical landmarks
+ all abnormalities
Los Angeles (reflux esophagitis)
Prague (Barrett's esophagus)
Baveno (varices)
Zargar (caustic esophagitis)
EREFS (eosinophilic esophagitis)
Forrest (bleeding ulcer)
Spigelman ((A)FAP or MAPS)
Paris (superficial suspected lesion)
Submucosal lesion (location, size, ulcus)
Seattle (4 per 2 cm, separate jars)
MAPS II (2 antrum, 2 corpus, separate jars)
EoE (≥ 4 biopsies, at least 2 levels)
Celiac disease (≥ 4 biopsies, at least 1 bulb)
Malignancy (≥ 4 biopsies)
Proximal esophagus
Distal esophagus
Z-line and diaphragm indentation
Cardia and fundus in inversion
Corpus in forward view including lesser curve
Corpus in retroflexion including greater curve
Angulus in partial retroflexion
Gastric antrum
Duodenal bulb (D1)
Second part of duodenum (D2)
Major papilla
Upper limit of the gastric folds
Eosinophilic esophagitis
Barrett's esophagus (Seattle protocol)
Premalignant stomach (MAPS II protocol)
Celiac disease
Radboudumc
