# Supplementary material for: Impact of a training intervention on upper gastrointestinal endoscopy quality over time: Multicenter comparative cohort study
Source: Endosc Int Open. 2025 Mar 14;13:a25260240. doi: 10.1055/a-2526-0240 (PMC11922177; doi:10.1055/a-2526-0240)
Supplement: Supplementary file 2 — Supplementary Material [file 10-1055-a-2526-0240_25294071.pdf]

eMethods

Table 1 Quality score, calculated as achieved points/maximum to be obtained (8 points) x 100%.

Inspection time		Photodocumentation*		Standardized terminology†		Biopsy guidelines‡	
≥ 7 min	2	≥ 10 landmarks and all abnormalities	2	Correct use of all applicable terminology or no use when not applicable	2	All biopsies taken when indicated or not taken when not indicated	2
5-7 min	1	6-9 landmarks and all abnormalities	1	Correct use of some but not all applicable standardized terminology	1	Partly taken	1
< 5 min	0	< 6 landmarks or no abnormalities	0	No use of terminology when applicable	0	No biopsies taken when indicated or taken when not indicated	0

*Proximal esophagus, distal oesophagus, squamocolumnar junction, upper end of the gastric folds, diaphragmatic indentation, retroflex fundus/cardia, corpus, angulus, antrum, duodenal bulb, distal duodenum, major papilla, all abnormalities.
†Los Angeles, Zargar, Prague, Forrest, Spigelman, Paris, Baveno, EREFS classification and description of submucosal lesions (location, size, ulcer on top).
‡Seattle, MAPS II, eosinophilic esophagitis, celiac disease, suspected neoplasia protocol.

<u>Criteria for awarding points are based on the following:</u>

Every upper gastrointestinal endoscopy should be able to (1) score the maximum amount of points and (2) lose points on all items based on performance.

Inspection time:
- 2 points for ≥ 7 minutes based on ESGE guideline [1]
- 1 point for 5-7 minutes based on a large retrospective study that showed similar neoplasia detection rates for 5-7 minutes inspection time compared to ≥ 7 minutes [2]

Photodocumentation:
- 2 points for ≥ 10 landmarks and all abnormalities based on ESGE guideline [1]
- 1 point for 6-9 landmarks and all abnormalities based on expert opinion

Standardized terminology:
- 2 points for correct use of all applicable terminology or no use when not applicable based on ESGE guideline [1]
- 1 point for correct use of some but not all applicable standardized terminology based on common sense

Biopsy guidelines:

- 2 points for all biopsies taken when indicated or not taken when not indicated based on ESGE guideline
- 1 point for partly biopsies taken when indicated based on common sense

1 *Bisschops R, Areia M, Coron E* et al. Performance measures for upper gastrointestinal endoscopy: a European Society of Gastrointestinal Endoscopy (ESGE) Quality Improvement Initiative. Endoscopy 2016; 48: 843-864
2 *Kawamura T, Wada H, Sakiyama N* et al. Examination time as a quality indicator of screening upper gastrointestinal endoscopy for asymptomatic examinees. Dig Endosc 2017; 29: 569-575

eResults

Table 2 Outcome of current upper gastrointestinal endoscopy*

		Overall (n = 570)	Before training (n = 285)	After training (n = 285)
**No significant pathology, n (%)**		**299 (53)**	**147 (52)**	**152 (53)**
	No pathology	155 (27)	92 (32)	63 (22)
	Diaphragmatic hernia	183 (32)	80 (28)	103 (36)
	Gastritis	76 (13)	27 (9)	49 (17)
	Cardia insufficiency	32 (6)	14 (5)	18 (6)
	Gastric erosion/erythema	26 (5)	15 (5)	11 (4)
	Duodenitis/bulbitis	23 (4)	12 (4)	11 (4)
	Reactive gastropathy	14 (2)	11 (4)	3 (1)
	Other	29 (5)	13 (5)	16 (6)
**Benign pathology, n (%)**		**151 (27)**	**80 (28)**	**71 (25)**
	Reflux esophagitis	87 (15)	40 (14)	47 (16)
	Gastric polyps†	67 (12)	32 (11)	35 (12)
	Varices	18 (3)	9 (3)	9 (3)
	Esophageal stenosis	17 (3)	12 (4)	5 (2)
	Candida esophagus	16 (3)	12 (4)	4 (1)
	Ulcus ventriculi	12 (2)	7 (3)	5 (2)
	Portal hypertensive gastropathy	11 (2)	4 (1)	7 (3)
	Esophageal polyp	5 (1)	2 (1)	3 (1)
	Angiodysplasia	5 (1)	1 (< 1)	4 (1)
	Eosinophilic esophagitis	5 (1)	1 (< 1)	4 (1)
	Other	28 (5)	13 (5)	15 (6)
**(Pre)malignant pathology, n (%)**		**120 (21)**	**58 (20)**	**62 (22)**
	H. pylori gastritis	40 (7)	23 (8)	17 (6)
	Gastric atrophy or intestinal metaplasia	38 (7)	18 (6)	20 (7)

	Barrett’s esophagus	32 (6)	15 (5)	17 (6)
	Duodenal polyps/adenomas	15 (3)	7 (2)	8 (3)
	Eesophageal cancer	8 (1)	5 (2)	3 (1)
	Gastric cancer	4 (1)	1 (< 1)	3 (1)
	Duodenal dysplasia	1 (< 1)	0 (0)	1 (< 1)
	Melanoma metastasis cardia	1 (< 1)	0 (0)	1 (< 1)
	Duodenal growth of pancreascarcinoma	1 (< 1)	0 (0)	1 (< 1)
	Extranodal B-cell lymphoma	1 (< 1)	1 (< 1)	0 (0)

*Multiple outcomes can exist simultaneously.
†Concerning all gastric polyps (incl. fundic gland polyps).

Table 3 Quality score and effect of training intervention.

	All (n = 570), mean (SD)	Before training (n = 285), mean (SD)	After training (n = 285), mean (SD)	Effect of training, % (95% CI)	*P* value for differences within variable on effect of training
**Center**					0.481
Center 1 (n = 190)	68 (16)	65 (17)	72 (14)	7.6 (3.2-11.9)	
Center 2 (n = 190)	59 (15)	56 (16)	61 (15)	5.1 (0.7-9.4)	
Center 3 (n = 190)	64 (19)	59 (18)	68 (17)	8.7 (4.3-10.2)	
**Procedure characteristics**					
**Urgency of endoscopy**					0.254
< 24 h (n = 45)	67 (17)	66 (18)	69 (17)	0.7 (-8.4-9.9)	
24-72 h (n = 21)	65 (19)	64 (21)	66 (18)	2.9 (-9.9-15.7)	
> 72 h (n = 504)	63 (17)	59 (17)	67 (16)	8.0 (5.3-10.7)	
**Type of sedation***					0.482
No sedation (n = 55)	59 (18)	55 (17)	66 (18)	8.6 (0.4-16.7)	
Topical pharyngeal anesthesia (n = 176)	62 (15)	60 (15)	65 (15)	4.4 (-0.2-9.0)	
Midazolam (n = 261)	66 (19)	61 (20)	69 (17)	9.0 (5.2-12.7)	
PSA or general anaesthesia (n = 41)	64 (15)	61 (16)	67 (14)	8.0 (-1.3-17.4)	
**Patient tolerance***					0.706
Good-fair (n = 275)	68 (16)	64 (17)	71 (15)	6.6 (3.1-10.0)	
Poor-very poor (n = 45)	63 (18)	60 (17)	67 (19)	4.8 (-3.8-13.4)	
**Indication**					0.571
Dyspepsia/reflux (n = 200)	64 (15)	61 (15)	67 (15)	5.7 (1.5-9.8)	
Anemia (n = 91)	60 (18)	57 (18)	63 (18)	7.6 (1.5-13.8)	
Dysphagia (n = 83)	63 (18)	59 (19)	66 (16)	7.9 (1.5-14.3)	
Hematemesis/melena (n = 36)	64 (20)	60 (20)	73 (17)	9.0 (-1.4-19.4)	
Surveillance (n = 33)	74 (16)	70 (16)	76 (16)	8.4 (-1.7-18.6)	
Abnormality on image (n = 24)	67 (18)	72 (27)	65 (11)	-4.5 (-17.2-8.2)	

Other (n = 103)	63 (18)	57 (17)	68 (16)	9.8 (4.0-15.5)	
**Outcome of endoscopy**					0.767
No significant pathology (n = 299)	63 (15)	59 (14)	66 (16)	6.2 (2.7-9.7)	
Benign pathology (n = 151)	63 (18)	59 (20)	68 (15)	8.3 (3.6-13.1)	
(Pre)malignant pathology (n = 120)	66 (20)	63 (21)	69 (19)	7.5 (2.2-12.9)	
**ENDOSCOPIST CHARACTERISTICS**					
**Endoscopist**					0.949
Endoscopist 1 (n = 6)	73 (9)	69 (9)	75 (10)	6.3 (-18.9-31.4)	
Endoscopist 2 (n = 22)	80 (15)	76 (16)	86 (12)	10.0 (-2.8-22.9)	
Endoscopist 3 (n = 4)	59 (12)	55 (7)	75 (n.a.)	20.8 (-12.7-54.4)	
Endoscopist 4 (n = 1)	63 (n.a.)	63 (n.a.)	n.a.	n.a.	
Endoscopist 5 (n = 6)	67 (19)	63 (n.a.)	68 (21)	5.0 (-26.8-36.8)	
Endoscopist 6 (n = 8)	63 (15)	61 (15)	75 (n.a.)	14.3 (-16.8-45.4)	
Endoscopist 7 (n = 5)	48 (14)	38 (n.a.)	50 (14)	12.5 (-20.0-45.0)	
Endoscopist 8 (n = 19)	74 (13)	66 (12)	76 (13)	10.2 (-6.1-26.6)	
Endoscopist 9 (n = 22)	64 (17)	58 (21)	67 (14)	9.0 (-3.6-21.6)	
Endoscopist 10 (n = 15)	66 (13)	68 (13)	63 (14)	-5.6 (-20.9-9.8)	
Endoscopist 11 (n = 40)	69 (16)	66 (19)	73 (12)	7.0 (-2.3-16.3)	
Endoscopist 12 (n = 24)	65 (12)	62 (14)	69 (7)	7.8 (-4.1-19.7)	
Endoscopist 13 (n = 18)	68 (20)	61 (23)	74 (17)	12.8 (-1.0-26.6)	
Endoscopist 14 (n = 10)	63 (20)	56 (21)	72 (21)	15.6 (-3.1-34.4)	
Endoscopist 15 (n = 38)	52 (12)	48 (11)	55 (11)	7.9 (-1.7-17.6)	
Endoscopist 16 (n = 23)	57 (15)	56 (15)	60 (16)	4.4 (-10.3-19.1)	
Endoscopist 17 (n = 26)	64 (20)	56 (23)	68 (18)	12.8 (0.9-24.8)	
Endoscopist 18 (n = 29)	57 (14)	67 (15)	67 (14)	-0.1 (-11.0-10.7)	
Endoscopist 19 (n = 18)	53 (13)	54 (14)	50 (0)	-4.5 (-20.9-12.0)	
Endoscopist 20 (n = 22)	59 (10)	60 (12)	58 (10)	-1.7 (-14.1-10.8)	
Endoscopist 21 (n = 13)	55 (15)	50 (9)	58 (18)	7.8 (-8.8-24.4)	
Endoscopist 22 (n = 11)	57 (15)	55 (19)	58 (13)	3.3 (-14.3-20.9)	
Endoscopist 23 (n = 57)	77 (12)	72 (12)	84 (9)	11.7 (4.0-19.5)	

Endoscopist 24 (n = 18)	63 (17)	59 (23)	66 (13)	7.0 (-7.1-21.0)	
Endoscopist 25 (n = 4)	59 (28)	56 (44)	63 (18)	6.3 (-22.8-35.3)	
Endoscopist 26 (n = 68)	50 (13)	48 (14)	54 (13)	5.5 (-1.7-12.7)	
Endoscopist 27 (n = 24)	67 (18)	62 (19)	74 (14)	12.3 (0.4-24.2)	
Endoscopist 28 (n = 19)	67 (19)	n.a.	67 (19)	n.a.	
**Gender of endoscopist**					0.483
Male (n = 323)	61 (18)	57 (18)	65 (17)	7.9 (4.5 - 11.3)	
Female (n = 247)	67 (16)	64 (16)	70 (15)	6.1 (2.4 - 9.9)	
**Age of endoscopist**	n.a.	n.a.	n.a.	0.0 (-0.3-0.4) per aging year	0.790
**Experience of endoscopist**					0.771
Resident (n = 120)	68 (15)	64 (17)	71 (12)	6.4 (1.0-11.9)	
Consultant (n = 450)	62 (18)	59 (17)	66 (17)	7.3 (4.5-10.2)	

ASA, American Society of Anesthesiologists; BMI, body mass index; n.a., not applicable; SD, standard deviation; UGI; upper gastrointestinal.
*Missing: BMI n = 84, alcohol n = 105, smoking n = 48, ASA n = 93, family history of UGI malignancy n = 283, daypart of endoscopy n = 1, use of sedation n = 37, type of sedation n = 37, patient tolerance n = 250.

Table 4 Use of terminology classifications.

Overall (n = 570)	Not used when not applicable	Used when applicable	Not used when applicable	Used when not applicable	n correct use/N applicable
**Forrest, n (%)**	554 (97)	8 (1)	8 (1)	0 (0)	8/16 (50)
**Prague, n (%)**	533 (94)	37 (7)	0 (0)	0 (0)	37/37 (100)
**Zargar, n (%)**	570 (100)	0 (0)	0 (0)	0 (0)	0/0 (NA)
**Spigelman, n (%)**	563 (99)	6 (1)	1 (<1)	0 (0)	6/7 (86)
**Paris, n (%)**	557 (98)	0 (0)	13 (2)	0 (0)	0/13 (100)
**Baveno, n (%)**	552 (97)	11 (2)	7 (1)	0 (0)	11/18 (61)
**Los Angeles, n (%)**	485 (85)	76 (13)	9 (2)	0 (0)	76/85 (89)
**EREFS, n (%)**	569 (100)	1 (< 1)	0 (0)	0 (0)	1/1 (100)
**Submucosal, n (%)**	560 (98)	2 (< 1)	8 (1)	0 (0)	2/10 (2)

Values are n (%) or n/N (%) unless otherwise defined.

Endosc Int Open | DOI 10.1055/a-2526-0240 | © The Author(s).

Thieme

Table 5 Use of biopsy protocols.

Overall (n = 570)	Not taken when not indicated	Taken when indicated	Not taken when indicated	Taken when not indicated	Some taken, but not completely when indicated	n correct use/N indicated
**Seattle, n (%)**	550 (97)	9 (2)	3 (1)	0 (0)	8 (1)	9/20 (45)
**EoE, n (%)**	504 (88)	36 (6)	19 (3)	1 (< 1)	10 (2)	36/65 (55)
**MAPS II, n (%)**	329 (58)	22 (4)	51 (9)	7 (1)	161 (28)	22/234 (9)
**Celiac, n (%)**	358 (63)	81 (14)	79 (14)	8 (2)	44 (8)	81/204 (40)
**Malignancy, n (%)**	556 (98)	11 (2)	0 (0)	0 (0)	3 (1)	11/14 (79)

Values are n (%) or n/N (%) unless otherwise defined.
EOE, eosinophilic esophagitis; MAPS, Management of Epithelial Precancerous Conditions and Lesions in the Stomach.

Table 6 Variables associated with higher quality scores.

	Univariable analysis			Multivariable analysis		
	P value	Effect, %	95% CI	*P* value	Effect, %	95% CI
**Gender of patient (male)**	0.009	3.4	0.9-5.9	0.012	3.2	0.7-5.7
**Age of patient, increment per year**	0.057	-0.1	-0.2-0.0			
**Alarming features (yes vs. no)**	0.013	-3.2	-5.7 to -0.7	0.017	-3.1	-5.6 to -0.5
**Daypart of endoscopy (afternoon vs. morning)**	0.706	-0.5	-3.3-2.3			
**Urgency of endoscopy**	0.210					
< 24 h		ref				
24-72 h		-1.8	-9.8-6.3			
> 72 h		-4.0	-8.7-0.7			
**Sedation**	0.426					
No sedation		ref				
Topical pharyngeal anesthesia		-0.1	-5.0-4.8			
Midazolam		1.2	-3.4-5.9			
PSA or general anesthesia		-3.0	-9.6-3.5			
**Indication**	0.085					
Dyspepsia/reflux		ref				
Anemia		-3.1	-6.9-0.8			
Dysphagia		-2.7	-6.6-1.2			
Hematemesis/melena		0.3	-5.1-5.8			
Surveillance		3.5	-2.3-9.4			
Abnormality on image		3.4	-3.1-9.9			
Other		-3.3	-7.1-0.4			
**Outcome of endoscopy**	0.390					
No significant pathology		ref				
Benign pathology		-1.8	-4.8-1.2			
(Pre)malignant pathology		0.5	-2.8-3.8			
**Gender of endoscopist (male)**	0.285	-3.4	-9.9-3.0			
**Age of endoscopist, increment per year**	0.021	-0.4	-0.8 to -0.1	0.028	-0.4	-0.8 to -0.1
**Experience of endoscopist (consultant vs. resident)**	0.208	-5.0	-13.0-3.0			

CI, confidence interval; PSA, procedural sedation and anesthesia.
